# Neuropelveology in Endometriosis: Pain Mechanisms, Diagnostic Implications, and Surgical Decision-Making

**DOI:** 10.3390/jcm15208177

**Published:** 2026-10-21

**Authors:** Cano-Herrera Gabriela, Ericka Cristina Loza López, Maria Fernanda Bautista Gonzalez, Felipe Esparza Salazar, Tamara Mena Guerrero, Diego De León Vela, Ximena A. van Tienhoven, Emmanuel Simental Aldaba, Patricia Carolina Massieu Pérez, Layra Mena García, Eder Gabriel Rivera Rosas, Dante Carbajal Ocampo, Ramiro Cabrera Carranco, Perla Sofía Vera Hernández, Axel Ranferi García Gaona, Jonathan Fidel Cueto Camara, Javier Iván Armenta Moreno, Ana Gabriela Sierra Brozon

**Affiliations:** 1 Escuela de Ciencias de la Salud, Universidad Anáhuac Puebla, Puebla 72810, Mexico; gabriela.canohe@anahuac.mx; 2 Centro de Investigación en Ciencias de la Salud (CICSA), Facultad de Ciencias de la Salud Universidad Anáhuac México Norte, Huixquilucan 52786, Mexico; cristyloza01@gmail.com (E.C.L.L.); mariafrenandabg19@gmail.com (M.F.B.G.); felipe.esparzas69@anahuac.mx (F.E.S.); tamara.menagu@gmail.com (T.M.G.);; 3 Department of Neurosurgery and Brain Repair, Center of Excellence for Aging and Brain Repair, Morsani College of Medicine, University of South Florida, Tampa, FL 33612, USA; 4 Centro de Rehabilitación e Inclusión Infantil Teletón Estado de México, Tlalnepantla 54010, Mexico; 5 Facultad de Medicina, Universidad Nacional Autónoma de México, Ciudad de México 04510, Mexico; 6 Facultad de Ciencias de la Salud, Universidad Panamericana, Ciudad de México 03920, Mexico; laymengar@gmail.com; 7 Female Pelvic Surgery Department, Doyenne High Quality and Multidisciplinary Treatment Center for Endometriosis, Ciudad de México 06700, Mexicoaxelranferi.gineco@gmail.com (A.R.G.G.);

**Keywords:** endometriosis, neuropelveology, chronic pelvic pain, neuroinflammation, pelvic nervous system

## Abstract

Endometriosis is a systemic, estrogen dependent neuroinflammatory-mediated disease affecting approximately 10–15% of women of reproductive age. Clinical manifestations include chronic pelvic pain (CPP), dysmenorrhea, dyspareunia, dyschezia, dysuria, and infertility. Endometriosis-associated pain is multifactorial and comprises overlapping nociceptive, neuropathic, and nociplastic pain phenotypes driven by persistent inflammation, neuroangiogenesis, peripheral nerve involvement, and central sensitization (CS). These mechanisms may sustain pain beyond lesion excision, highlighting the importance of pelvic nervous system (PNS) involvement. This narrative review examines the role of neuropelveology (NP) in endometriosis, focusing on pain mechanisms, diagnostic implications, and surgical decision making. NP integrates pelvic neuroanatomy and neurophysiology into structured neurological assessment, pain mapping, symptom localization, and nerve sparing surgery. Current evidence suggests that NP may complement conventional evaluation in selected patients with refractory pain, neuropathic symptoms, discordance between symptom severity and imaging findings, or suspected involvement of the sacral plexus or sciatic nerve. Recognizing distinct pain phenotypes may support individualized treatment planning by balancing lesion excision with neural preservation and identifying patients who may benefit from multidisciplinary pain management, rehabilitation, neurolysis, or neuromodulation. Although current evidence remains limited, NP should be considered a complementary framework rather than a replacement for established endometriosis management. Prospective multicenter studies using standardized diagnostic criteria and long-term functional outcomes are needed to define its clinical utility.

## 1. Introduction

Neuropelveology (NP) is an emerging interdisciplinary subspecialty dedicated to the study of pelvic neuroanatomy and neurophysiology to evaluate and manage disorders involving the pelvic nervous system (PNS) [1]. By combining anatomical, functional, and surgical perspectives, NP provides a comprehensive framework for the diagnosis and treatment of sensory, motor, and autonomic dysfunctions arising from pelvic nerve injury or disease. Growing evidence suggests NP as a promising tool in the approach and management of PNS involvement in chronic pelvic conditions. The most common pathologies involving the sacral plexus treated using NP are radiculopathies, compressions, deep infiltrating endometriosis (DIE) and tumors [1,2]. Among gynecological causes, endometriosis remains one of the most common etiologies, affecting approximately 10–15% of women of reproductive age, with 35–50% of affected individuals experiencing CPP during the course of the disease [3].

The development of NP has been largely driven by the need to better understand and manage CPP, a complex condition affecting approximately 15–26% of women worldwide and representing one of the most prevalent and debilitating gynecological diseases [3,4]. CPP is associated with a substantial reduction in quality of life, sexual function, psychological well-being, and daily functioning. CPP may arise from a wide spectrum of identifiable pelvic and extrapelvic disorders; however, in some patients an underlying etiology may remain unclear after the initial diagnostic evaluation, highlighting the multifactorial nature of CPP, limitations of the current diagnostic evaluations [5].

Increasing evidence indicates that CPP shares pathophysiological characteristics with other chronic overlapping pain conditions, particularly alterations in central pain processing pathways [6,7]. Sustained nociceptive stimulation may induce maladaptive neuroplastic changes within the central nervous system (CNS) at both spinal and supraspinal levels, resulting in enhanced pain perception and the development of nociplastic pain. These mechanisms may contribute to the persistence, amplification, and multidimensional nature of pain observed in many patients with endometriosis, even after apparently successful treatment [4].

Traditional approaches to endometriosis have primarily focused on lesion detection, classification, and excision, often overlooking the complex interaction between the disease and the PNS [8]. Advances in the understanding of neuroimmune interactions, neural infiltration, and pain sensitization have highlighted the value of NP as a complementary framework [2]. This narrative review examines the role of NP in endometriosis, with particular emphasis on pain mechanisms, improved diagnostic accuracy, and its contribution to surgical management strategies. This review assesses: (1) nociceptive, neuropathic, and nociplastic mechanisms associated with pain in endometriosis and the pelvic nervous system. (2) NP potential approach to diagnosis and nerve involvement in pain. (3) Implications of nerve involvement and pain phenotyping applied to patient selection, surgical management and nerve-preserving strategies. The review addressed current limitations in the literature and future perspectives on the clinical role of NP in endometriosis.

## 2. Methods

A comprehensive literature search was conducted using PubMed, EBSCO, Cochrane, Google Scholar. The search strategy included a combination of Medical Subject Headings (MeSH) including “endometriosis”, “endometrioses”, “endometrioma”, “endometriomas”, “endometriosis/classification”, “endometriosis/diagnosis”, “endometirosis/surgery”, endometriosis/rehabilitation”, “pelvis/innervation”, “pelvis/phyisiology”, “pelvis/physiopathology”, “pelvis/surgery”, pelvis/pathology”, “peripheral nerves”, “pelvic pain”, “pelvic inflammatory disease” pudendal neuralgia”, peripheral nerve injury”, “neuroinflammatory diseases”. We also included terms such as “Neuropelveology”, “LION”, “Laparoscopic Implantation of Neuroprosthesis” and “Deep Infiltrating Endometriosis” in the search strategy. The initial database search allowed a preliminary selection of key articles, from which we identified specific studies through the snowball method. This method allowed us to identify key neuropelveology articles in the literature.

This narrative review considered scientific articles addressing at least one of our predefined domains: (1) pain mechanisms and nerve involvement in endometriosis, (2) NP diagnostic evaluation, (3) therapeutic and surgical implications of NP in endometriosis. We included randomized controlled trials, observational studies, systematic reviews, meta-analyses, narrative reviews, and experimental animal studies. We applied no language or year-of-publication restrictions. We screened articles based on title, abstract, and full text. A total of 56 articles were ultimately included in this review.

## 3. Pain Mechanisms in Endometriosis and the Neuropelveological Approach

### 3.1. Generalities of Endometriosis

Endometriosis is a common yet underrecognized estrogen dependent systemic disease that affects approximately 10–15% of women in reproductive age (25–45 years). The incidence increases significantly to 50% among women experiencing infertility and ranges between 35–50% in those presenting with CPP [3,9].

The disease is characterized by the growth of endometrial-like tissue, comprising endometrial glands and stroma, outside the uterine cavity. Pelvic organs are the most commonly affected sites, although it may also involve extrapelvic sites containing mesothelium, such as the pleura, peritoneum, and pericardium. This ectopic implantation is associated with systemic inflammatory responses and clinical manifestations including CPP, dyspareunia, infertility, dysmenorrhea, dyschezia, and hematochezia [9,10,11].

The etiology of endometriosis continues to be a condition whose causes and progression are not fully understood and is currently considered multifactorial, involving immune, endocrine, and inflammatory processes [12]. Sampson’s retrograde menstruation theory proposes that menstrual blood containing viable endometrial cells may reflux through patent fallopian tubes into the peritoneal cavity, implanting onto pelvic surfaces, where they proliferate, and form lesions. Even though this remains one of the most widely recognized hypotheses, it fails to sufficiently explain certain clinical presentations, such as DIE, extrapelvic endometriosis, cases reported in premenarchal girls, and in male subjects [9,12,13].

In contrast to theories centered on endometrial shedding and retrograde flow, alternative models suggest an embryological origin for ectopic tissue. Müllerianosis has been described as the presence of residual mullerian tissue within ectopic cavities resulting from aberrant migration or differentiation during embryogenesis, potentially leading to endometriosis [14]. Similarly, the coelomic metaplasia theory proposes that abdominal mesothelial cells retain the capacity to transform into endometrial-like tissue in response to stimuli such as chronic inflammation [13]. This mechanism could account for the presence of lesions in anatomical regions where menstrual reflux is physiologically impossible [12,15].

Modern recent frameworks emphasize that endometriosis pathogenesis is not restricted to a single isolated mechanism, but rather results from the convergence of multiple pathways, including menstrual reflux, immune dysfunction, metaplasia, cellular migration mechanisms, and both local and systemic hormonal alterations [16]. In this context, the establishment and progression of lesions are thought to require a permissive systemic microenvironment characterized by persistent inflammation, oxidative stress, and immune dysregulation, while CPP may develop in parallel through neuroimmune mechanisms [17].

### 3.2. Chronic Pelvic Pain

CPP is pain perceived by the pelvic structures and is characterized by being non-malignant [18]. The condition arises from a wide spectrum of pelvic and extrapelvic disorders, including hemorrhoidal disease, irritable bowel syndrome, pudendal neuropathy, sacral spinal cord injuries, vascular disorders, infections, anorectal conditions and endometriosis [5,18]. Therefore, CPP may benefit from the multidisciplinary approach offered by NP in order to provide a more comprehensive medical care [19].

Traditionally, CPP has been associated with an identifiable underlying pathology, however, pain may persist despite adequate medical management or complete surgical treatment of the primary disease. When pain persists for more than six months following adequate treatment of the presumed primary condition, it is considered that Chronic Pelvic Pain Syndrome (CPPS) has developed [4,5].

#### 3.2.1. Chronic Pelvic Pain in Endometriosis

Currently, conservative surgical treatment of endometriosis presents significant limitations due to the lack of complete clinical resolution in a substantial proportion of patients, surgical treatment of endometriosis presents limitations due to incomplete pain resolution in a subset of patients. Orr [7] reported that severe chronic pelvic pain persisted in 23.4% of 239 patients after surgery for endometriosis, with a mean follow-up of 16.1 months. In addition, 36.8% showed no clinically meaningful improvement, while 8.4% experienced worsening pain. Endometriosis-associated pain is driven by a complex interaction between local nociceptive signaling and a sustained inflammatory microenvironment. Multiple mechanisms including peripheral, central, and cross-sensitization, lesion-specific molecular alterations, and psychosocial factors may contribute to the development and maintenance of CPP [6,18,20].

CPP establishment depends on neuroangiogenesis, a process characterized by coordinated neovascularization and sensory nerve fiber sprouting that promotes aberrant innervation of endometriotic lesions and facilitates persistent nociceptive transmission [6,18,21]. Importantly, the severity of pain correlates poorly with lesion burden or disease stage, and pain often persists or recurs following apparently complete surgical excision, highlighting the contribution of sustained neuroinflammation and central sensitization (CS) [7,20,22].

Endometriotic implants may develop throughout the pelvis and infiltrate somatic and autonomic nerves, particularly the sacral plexus and sciatic nerve, producing neuropathic pain manifestations [21,23,24]. Evidence for intraneural endometriosis is limited and is based mainly on case reports and small clinical series. However, isolated intraneural disease without concomitant peritoneal endometriosis has been described, suggesting that perineural dissemination may represent an additional, although still unproven, pathogenic mechanism [23]. Moreover, reports of intraneural endometriosis in the absence of peritoneal disease support perineural dissemination as an additional pathogenic mechanism [21]. The ectopic endometrial microenvironment is characterized by a sustained inflammatory milieu enriched with IL-1β, IL-6, TNF-α, prostaglandins, chemokines, NGF, and BDNF. These mediators sensitize peripheral nociceptors, promote peripheral sensitization, and establish a feed-forward inflammatory loop that perpetuates pain signaling [6]. Although ectopic endometrial lesions initially lack an organized sensory innervation, the acquisition of newly formed sensory nerve fibers through neuroangiogenesis establishes functional nociceptive pathways that sustain chronic pain transmission [20].

#### 3.2.2. Pain Phenotypes

Endometriosis-associated pain encompasses nociceptive, neuropathic, and nociplastic mechanisms, which often coexist and contribute to the heterogeneity of symptoms observed among affected women [21,25,26] Table 1.

##### Nociceptive Pain

Nociceptive pain results from the direct activation of peripheral nociceptors by ongoing tissue damage or inflammation, such as that produced by ectopic endometriotic lesions, peritoneal irritation, or mechanical distension of pelvic structures) [25]. In endometriosis, inflammatory mediators released by ectopic lesions, including cytokines, prostaglandins, and neurotrophic factors, stimulate nociceptive pathways and contribute to pain generation [6,27]. Clinically, it manifests as cyclical or activity-related pain closely correlated with the presence and activity of visible disease, including dysmenorrhea, dyspareunia, dyschezia, dysuria, and CPP [18,21,27].

##### Neuropathic Pain

Neuropathic pain results from a lesion or disease affecting the somatosensory nervous system (SNS) [25]. Particularly, DIE may involve pelvic nerves directly, specifically the sacral plexus and sciatic nerve, leading to neuropathic symptoms such as radiating pain, paraesthesia, sensory disturbances, and, less frequently, motor deficits [2,23]. Evidence of intraneural endometriosis and perineural spread supports the concept that neural infiltration may represent an independent mechanism of pain generation in some patients [23]. Neuropathic pain should therefore be considered in women who present with cyclical neuropathic symptoms or pain extending beyond the pelvis [2,23].

##### Nociplastic Pain

Nociplastic pain constitutes a third mechanistic category of chronic pain, distinct from nociceptive pain (caused by active tissue damage) and neuropathic pain (secondary to damage to the peripheral or central nervous system) [26,28]. This pain phenotype results from altered nociceptive processing despite the absence of sufficient peripheral tissue damage or a demonstrable lesion of the somatosensory nervous system to explain symptom severity [25]. CS is considered the primary mechanism underlying this condition, involving amplification of pain signaling within the CNS and enhanced responsiveness of spinal and supraspinal nociceptive pathways, which together produce pain hypersensitivity [22,25,26,29].

This alteration operates at multiple levels of the nervous system: at the peripheral level, subtle changes such as nociceptor sensitization and expansion of receptive fields may coexist; at the spinal level, spinal cord reorganization, wind-up facilitation, and glial activation have been described; and at the supraspinal level, hyperactivity and increased connectivity are observed between brain regions involved in pain and emotion processing, along with reduced activity in descending analgesic pathways. Clinically, this mechanism manifests as multifocal pain that is more intense or diffuse than would be expected given the degree of identifiable tissue or nerve damage and is often accompanied by associated symptoms of central origin, such as fatigue, sleep disturbances, cognitive dysfunction, and hypersensitivity to sensory stimuli [28].

According to Nijs [30], CS should be understood as a process composed of multiple mechanisms, in which there is an amplification of neural signaling within the CNS, clinically manifesting as pain hypersensitivity. This process may initially serve a protective purpose in response to tissue injury, such as surgery or an accident, and can therefore be considered a homeostatic event similar to inflammation. However, when it persists, it loses its adaptive function and becomes a maladaptive phenomenon [27]. Therefore, CS provides a framework for therapeutic approaches targeting the neurobiological mechanisms involved in pain maintenance [22,25,29,31,32]. Several validated clinical tools allow indirect assessment of CS. The CSI is a widely used self-report instrument that assesses CS related symptoms, with a score ≥ 40 suggesting the likely presence of CS [33,34]. Short forms of the CSI have also been developed to reduce patient burden while maintaining its clinical utility [35,36]. Quantitative sensory testing (QST) provides psychophysical measures of altered pain processing, including pressure pain thresholds, temporal summation, and conditioned pain modulation [37,38,39]. Because these approaches assess partially distinct aspects of pain sensitization, their combined use may provide a more comprehensive assessment of CS, with evidence demonstrating significant associations between CSI scores and QST measures [40,41]. Therefore, CS provides a framework for therapeutic approaches targeting the neurobiological mechanisms involved in pain maintenance [22,25]. In a systematic review, Zheng [22] describes how pain in endometriosis is associated with an abnormal angiogenic and neurogenic environment characterized by the proliferation of nerve fibers surrounding ectopic lesions.

In a systematic review, Zheng [22] describes how pain in endometriosis is associated with an abnormal angiogenic and neurogenic environment characterized by the proliferation of nerve fibers surrounding ectopic lesions. These fibers, exposed to a persistent inflammatory microenvironment, generate sustained nociceptive stimulation that induces plastic changes in peripheral synapses. This signaling is transmitted through the dorsal horn of the spinal cord to higher central structures, including the thalamus, brainstem, and cerebral cortex, where long-term alterations in synaptic transmission and glial cell function may occur [20,22]. Several clinical tools are available to assess central sensitization in this population. The Central Sensitization Inventory (CSI) is a validated 25-item self-reported questionnaire (scored 0–100) that assesses systemic pain and related symptoms; a cutoff ≥ 40 has demonstrated 78% sensitivity and 80% specificity for identifying patients with endometriosis and central sensitivity syndromes [42] QST provides objective neurophysiological measures including pressure pain thresholds and temporal summation of pain, though it requires specialized equipment [43]. Functional MRI and other neuroimaging approaches can identify altered brain connectivity in regions including the anterior cingulate cortex, thalamus, and hippocampus [44].

In this context, afferent and central pathways are amplified, generating hypersensitivity not only to painful or musculoskeletal stimuli, but also to sensory stimuli such as odors, light, sound, temperature, touch, stress, or chemical substances. Therefore, CS should not be understood solely as a pathophysiological concept, but also as a clinical tool that allows the identification of patients with persistent pain, expansion of the painful area, or symptoms disproportionate to the visible tissue damage, thereby guiding a more comprehensive assessment of chronic pain [30].

In endometriosis, persistent nociceptive input from endometriotic lesions may sustain CS, contributing to CPP despite surgical treatment and converging chronic pain syndromes [18,20,22,25,29]. However, it is important to acknowledge that a considerable portion of the mechanistic evidence supporting this hypothesis derives from animal models, and clinical studies in humans have been conducted in specific populations with heterogeneous methodologies. Therefore, although the CS hypothesis is biologically plausible and clinically relevant, further research is needed to fully elucidate the neurophysiological mechanisms involved and their translational implications in clinical practice [22].

#### 3.2.3. Neural Involvement and Neuroplasticity in Endometriosis

The neuroinflammatory nature of endometriosis secondary to the dynamic of the ectopic endometrial lesions and their interactions with peripheral nerves, immune cells, and the local vascular network, ultimately promotes CPP [20]. The ectopic microenvironment is enriched with neurotrophic factors, inflammatory cytokines, prostaglandins, and estrogens that collectively drive neuroplastic changes within and surrounding endometriotic lesions. Among these mediators, NGF plays a central role in promoting sensory nerve growth, whereas macrophages represent a major source of VEGF, inflammatory cytokines, and additional neurotrophic mediators that amplify neuroimmune crosstalk and support lesion vascularization, neural remodeling, and peripheral sensitization [20,45].

Ectopic lesions actively recruit and polarize macrophages, establishing a self-perpetuating inflammatory microenvironment that promotes lesion survival, angiogenesis, and aberrant nerve fiber growth [20,46]. In human endometriosis, peritoneal fluid analyses demonstrate elevated macrophage populations with M2 polarization that facilitate lesion vascularization, and murine models confirm that M2 macrophage injection enhances ectopic lesion growth whereas M1 macrophages are protective [47,48]. Histological studies in human endometriosis lesion consistently demonstrate an increased density of small unmyelinated sensory fibers together with sympathetic and parasympathetic nerve fibers within endometriotic implants, indicating that these lesions function as active neuroimmune interfaces rather than passive ectopic tissue [20,21,46,49,50]. Baboon models further demonstrate that nerve fiber density increases progressively over 12 months following lesion induction, suggesting temporal evolution of neurogenesis [51]. In parallel, elevated expression of NGF, and BDNF in human endometriotic tissue and peritoneal fluid stimulates sensory nerve sprouting and nociceptor sensitization, with IL-1β-mediated BDNF production confirmed in primary human endometrial stromal cell cultures, generating hyperinnervated lesions that provide persistent nociceptive input and facilitate the transition from cyclical pain to CPP [52,53,54].

Neuroangiogenesis constitutes a pivotal mechanism linking inflammation and pain in endometriosis. Coordinated angiogenesis and sensory nerve fiber sprouting promote the establishment of functional nociceptive pathways within ectopic lesions. Once established, these newly formed sensory fibers perpetuate a positive feedback process known as neurogenic inflammation [20,55]. Activation of peripheral nociceptors induce the release of neuropeptides that recruit and activate mast cells and macrophages, resulting in sustained production of pro-inflammatory cytokines, including TNF-α and IL-1β. This reciprocal interaction continuously reinforces inflammation, neuronal sensitization, and persistent pain signaling [20,55,56,57].

Persistent peripheral nociceptive stimulation ultimately induces maladaptive plasticity within the CNS. Sustained activation of primary afferent neurons increases excitability of dorsal horn neurons, promotes synaptic remodeling and axonal sprouting, and amplifies nociceptive transmission within spinal and supraspinal pain circuits [18,20]. Consequently, CS develops, allowing pain hypersensitivity to persist even after resolution of the initial peripheral stimulus or surgical excision of lesions. This persistent neuroplastic state is considered a major mechanism underlying pain chronification and treatment resistance in women with endometriosis [18,58].

Furthermore, recent neuroimaging studies have suggested that endometriosis-associated pain may be accompanied by structural changes in brain regions involved in pain processing, anticipation, memory, and emotional modulation. Maulitz [59] found differences in gray matter volume and cortical thickness in patients with endometriosis, particularly in regions such as the cerebellum, lingual gyrus, and calcarine gyrus, supporting the hypothesis that persistent pain may be related to processes of central neuroplasticity.

### 3.3. Introduction to Neuropelveology

NP integrates principles from neurology, gynecology, urology, colorectal surgery, and pain medicine to provide a comprehensive understanding of pelvic nerve function and pathology. The field was formally established in the early twenty-first century with the creation of the International Society of Neuropelveology by Dr. Marc Possover, with the objective of consolidating previously fragmented expertise regarding pelvic nerve disorders, their diagnostic evaluation, and therapeutic management [1]. Historically, pelvic nerves were primarily considered in the setting of surgical complications and neuropathic pain syndromes. However, only in recent years has a structured and systematic framework been developed to address the intricate functional and anatomical organization of the PNS [1,2,3].

The clinical applications of NP can be broadly categorized into four principal domains: neurological diagnosis through symptom guided localization of nerve lesions; functional assessment of somatic and autonomic pelvic pathways; surgical planning aimed at preserving neural structures and optimizing outcomes; and therapeutic interventions targeting pelvic nerve disorders through microsurgical, neuromodulatory, or rehabilitative approaches [1,5,60]. These applications have gained clinical relevance as the contribution of neural mechanisms to pelvic pain and dysfunction become better recognized [60].

The conceptual foundation of NP involves precise mapping of pelvic neural networks, clarification of their functional roles, and translation of this knowledge into clinical practice. The neurofunctional anatomy of the pelvis is defined by the complex dynamic between somatic, autonomic, and visceral components of the PNS, acknowledging the substantial overlap and bidirectional communication among these pathways [1,61] (Table 2). The clinical scope of NP extends to the management of pelvic neuropathies, lower urinary tract dysfunction, sexual dysfunction, anorectal disorders, and CPPS. In addition, it supports the development of nerve sparing surgical strategies and advances in neuromodulation therapies aimed at restoring or preserving pelvic organ function [1,61].

### 3.4. Somatic Innervation

Somatic innervation is predominantly mediated by the pudendal nerve, which originates from the ventral rami of S2 to S4 and provides motor and sensory supply to the perineum, external anal sphincter, and external urethral sphincter [62]. The sacral plexus, formed by contributions from the lumbosacral trunk and nerve roots S1 to S4, further supports motor and sensory innervation of the pelvic floor and portions of the lower extremities [63]. Variations in the course, branching pattern, and anatomical relationships of the pudendal nerve are clinically relevant, as they may predispose patients to entrapment syndromes or increase the risk of iatrogenic injury during pelvic surgical procedures [62,63,64].

#### 3.4.1. Autonomic Innervation

Autonomic innervation of the pelvis involves coordinated sympathetic and parasympathetic pathways. The superior hypogastric plexus, situated anterior to the aortic bifurcation and sacral promontory, gives rise to the hypogastric nerves, which descend bilaterally into the pelvis and contribute to the formation of the inferior hypogastric plexus [47,48,49,50,51,52,53,54,55,56,57,58,59,60,61,62,63,64,65]. The inferior hypogastric plexus is composed of sympathetic fibers from the hypogastric nerves, parasympathetic fibers from the pelvic splanchnic nerves arising from S2 to S4, and contributions from the sacral sympathetic trunk [65,66,67]. This plexus delivers autonomic innervation to the bladder, rectum, uterus, vagina, and other pelvic viscera, and demonstrates substantial interindividual variability in its structure and fiber distribution [65,66,67]. The presence of mixed cholinergic and adrenergic ganglia within the plexus further highlights the complexity of autonomic regulation in the pelvis [65,68].

#### 3.4.2. Visceral Innervation

Afferent and efferent pathways from the visceral innervation system are essential for coordinated pelvic organ function. Afferent fibers convey sensory input from pelvic viscera to the CNS, whereas efferent fibers mediate motor, secretory, and vascular responses. These pathways play a central role in the regulation of urinary, anorectal, and sexual function, and their disruption may result in neuropathic pain, incontinence, or visceral dysfunction [61,66]. The small caliber and dense anatomical arrangement of pelvic nerve fibers render them particularly vulnerable to injury secondary to obstetric trauma, spinal cord lesions, or surgical interventions, with clinical manifestations that may be delayed or clinically subtle [61,66].

The clinical relevance of pelvic neuroanatomy offered by NP is particularly evident in gynecology, urology, colorectal surgery, and the multidisciplinary management of CPPS [1,2]. Detailed anatomical knowledge is fundamental for the development and application of neuroprotective surgical techniques aimed at preserving nerve integrity and minimizing postoperative functional impairment [1,69].

## 4. Implications for Diagnosis

**Figure 1:**
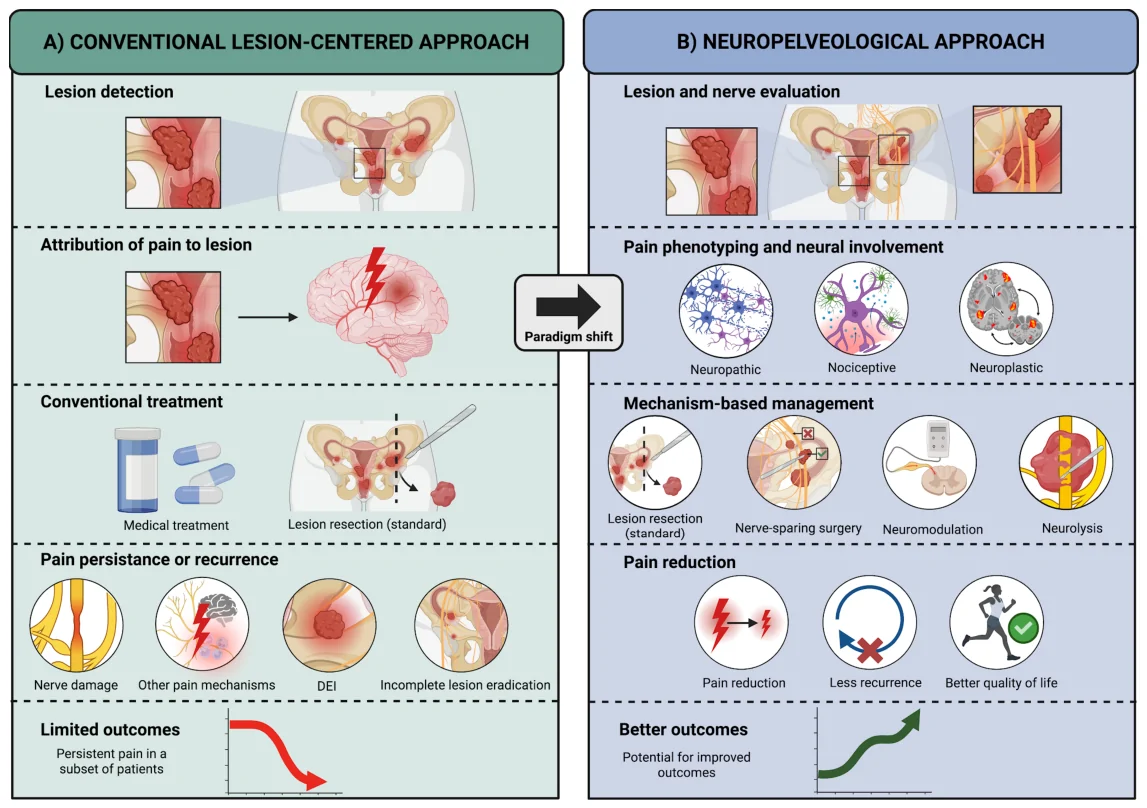
From lesion-centered management to a neuropelveological framework for endometriosis-associated pain.

The integration of NP into the diagnostic evaluation of endometriosis-associated pain represents a promising, but not yet universally established approach. Although it is not currently recognized as a standard diagnostic modality, growing evidence suggests that a neuropelveologic perspective may serve as a valuable complementary tool within a multidisciplinary framework, particularly in patients with complex or refractory symptoms [2,70] (Figure 1).

Endometriosis-related pain is increasingly understood as a multifactorial phenomenon that extends beyond purely inflammatory or mechanical mechanisms alone. Neural infiltration, irritation, and entrapment of somatic, autonomic, and visceral pelvic nerves have been implicated in the persistence, severity, and heterogeneity of symptoms [71]. Conventional imaging modalities such as transvaginal ultrasound (TVUS) and magnetic resonance imaging (MRI) are highly useful for detecting and mapping endometriosis. For DIE overall, MRI has demonstrated pooled sensitivity and specificity of approximately 83–94%, respectively, although diagnosis performances vary substantially according to the anatomical compartment evaluated [72]. More recent evidence has similarly demonstrated high diagnostic performance for several ENZIAN compartments, including the ovaries, tubo-ovarian region, vaginal/rectovaginal space, uterosacral ligaments, and rectosigmoid, whereas data remain insufficient for some other anatomical sites [72,73]. Currently, robust sensitivity and specificity estimates for MRI or TVUS in detecting microscopic neural infiltration, perineural spread or functional pelvic nerve dysfunction are lacking [2,70]. Therefore, negative or inconclusive imagining should not necessarily exclude neural involvement when clinical findings suggest a neuropathic phenotype [7].

A neuropelveologic assessment emphasizes the systematic evaluation of pelvic nerve pathways, including the pudendal nerve, sacral plexus, and autonomic hypogastric plexuses. Incorporating targeted neurological examination, pain mapping, and careful correlation of symptoms with known neuroanatomical distributions may enhance diagnostic precision in selected cases [1]. This approach is particularly relevant when clinical presentation suggests neuropathic characteristics such as burning pain, electric shock sensations, allodynia, or radiation along specific dermatomes [70]. This approach may also be of use in rare but clinically significant scenarios such as isolated DIE of sacral nerve roots, where the symptoms may overlap with sciatica. Failure to detect and address such involvement may result not only in persistent severe pain, but also in progressive and potentially irreversible neural damage [74].

Furthermore, CPP in patients with suspected or confirmed endometriosis frequently overlaps with other conditions that share neuropathic or functional features. Failure to recognize these entities may contribute to persistent symptoms after standard surgical or hormonal treatment [2,70,75]. Neural involvement also influences lower back pain, lower extremity symptoms, and pain patterns that mimic orthopedic or neurological disorders, frequently delaying diagnosis and treatment [2,23]. A neuropelveologic framework encourages a broader diagnostic lens, reducing the risk of attributing all pelvic pain exclusively to endometriotic lesions [75].

The potential utility of NP becomes particularly evident in complex clinical scenarios, including recurrent pain following surgical excision, discordance between symptom severity and imaging findings, or suspicion of nerve entrapment by DIE. In such cases, a structured evaluation of pelvic neural structures may assist in refining surgical planning and avoiding incomplete treatment or unnecessary reintervention [2,70].

In practical terms, neuropelveological assessment may inform surgical decision-making in several clinically relevant scenarios. In patients with DIE involving the sacral plexus, sciatic nerve, or other somatic nerves, recognition of the affected neural structure and the pattern of neurological involvement may support referral to specialized teams and guide the selection of nerve directed procedures such as decompression, neurolysis or excision. The recent literature emphasizes the importance of identifying pelvic nerves during surgery to reduce iatrogenic injury and preserve autonomic function, while nerve-sparing approaches have been associated with lower rates of postoperative voiding dysfunction and preservation of bowel and sexual function [70,76]. In patients with symptomatic sacral plexus endometriosis, surgical resection has been associated with improvement in neurological and pain symptoms, although substantial surgical morbidity has also been reported, emphasizing the need for individualized preoperative assessment and surgical planning [77]. Conversely, when severe pain is accompanied by features suggestive of nociplastic pain or central sensitization that are not fully explained by the anatomical extent of disease, assessment of pain mechanisms may support consideration of multidisciplinary pain management rather than relying exclusively on further lesion directed surgery [25].

Despite its conceptual strengths, NP remains an evolving discipline, and high-quality prospective data validating standardized diagnostic protocols are limited. Therefore, it should currently be regarded as a complementary diagnostic approach rather than a replacement for established imaging and surgical evaluation. Further research is needed to define objective diagnostic criteria, optimize patient selection, and clarify its impact on long term outcomes.

## 5. Surgical Decision-Making

Surgical approaches for endometriosis usually bring symptom relief and improvement of pain. However, persistent or recurrent pain after eradication surgery is frequently reported [6,75]. Prolonged nociceptive input from endometriotic lesions can remodel pain processing pathways and promote persistent hypersensitivity, contributing to CPP even after lesion removal [22]. Residual pain syndromes in these patients highlight the complexity of CPP, suggesting that lesion eradication alone may be insufficient, thus requiring more comprehensive approaches [75,78,79].

Traditional surgical planning in endometriosis is primarily lesion-oriented and anatomy-based, often without systematic consideration of pelvic neural pathways involved in pain generation [79]. Emerging evidence suggests that selected neuropelveological interventions, including neurolysis, neurectomy, and neuromodulation, may improve outcomes in carefully selected patients with neural involvement [2]. Recognizing the neuroinflammatory and neuroplastic components of endometriosis-associated pain may therefore improve diagnostic accuracy, optimize treatment selection, and support more personalized management strategies [25].

Standard surgery’s primary objective is to eradicate the macroscopically visible endometriotic tissue. Despite this, such an approach does not guarantee complete disease resolution, as microscopic disease, non-resectable lesions or alternative pain mechanisms may persist, including neuropathic and centrally mediated mechanisms [75,78]. The heterogeneous nature of pain related to endometriosis highlights the need for individualized pain phenotyping and comprehensive neurological assessment. Patients often present with overlapping nociceptive, neuropathic, and nociplastic mechanisms that may respond differently to medical and surgical interventions [25]. NP offers a framework for identifying pelvic nerve dysfunction and integrating neuroanatomical, neurological, and surgical perspectives into patient care [5].

Pelvic laparoscopic surgery has become the standard treatment for endometriosis, nevertheless, postoperative pain remains a frequent and poorly characterized outcome, which may result in long-term functional morbidities. High rates of pain recurrence and reoperation have been reported even after conservative or definitive surgical approaches. This issue challenges the traditional therapeutic model and highlights important gaps in the comprehensive understanding of the disease’s pathophysiology [1]. It is important to distinguish between pain recurrence and persistent postoperative pain. Persistent pain refers to symptoms that continue after surgery without a meaningful pain-free interval and may reflect ongoing nociceptive input, peripheral nerve involvement, or other pain-generating mechanisms, even in the absence of detectable residual disease. In contrast, pain recurrence refers to the reappearance of symptoms after an initial period of postoperative improvement and may be associated with new lesions, disease recurrence, or reactivation of nociceptive pathways. This distinction is clinically relevant because persistent pain, particularly when predominantly driven by central sensitization, may not respond to repeated lesion-directed surgical interventions [6,23]. Orr [7] and Roman [80] reported 23% and 44.6% of patients who underwent a second surgery after a 7-year follow-up period, respectively. Fertility sparing surgery has been identified as a risk factor to endure recurrent pain and reintervention, suggesting that pain mechanisms beyond the endometriotic lesions themselves may be involved [7].

Alboni [75] conducted a retrospective cohort analysis in which, out of 345 patients who underwent surgery for endometriosis, 97 patients (28.1%), reported persistent post-operative pain after 3 months, despite the absence of detectable endometriotic lesions. These findings support the involvement of neural pathways in the pathophysiology of endometriosis-related pain, reinforcing the hypothesis that pain persistence may occur independently of residual or recurrent disease. Understanding endometriosis as a systemic and multifactorial condition involving nociceptive, neuropathic, and neuroplastic pain mechanisms is essential to reduce long-term morbidity and offer patients a better quality of life [1].

Becker [81] analyzed pain recurrence after endometriosis treatment in a systematic review including 3198 patients who underwent surgery followed by medical therapy between 1992 and 2014. A median of 31% of patients reported persistent pain at the end of medical treatment, leading authors to suggest that surgical factors, such as adhesions or nerve-related injury, may contribute to postoperative pain persistence. In this context, surgical decision-making must balance lesion resection against neural preservation, particularly in cases where pain patterns suggest neuropathic involvement. In practice, achieving this balance begins with the preoperative identification of patients in whom imaging findings or clinical symptoms suggest involvement of the major pelvic neural structures. Surgical planning should consider the anatomical relationship between endometriotic lesions and the sacral plexus, pudendal nerve, sciatic nerve, and pelvic autonomic pathways, together with the potential functional consequences of nerve injury [82]. When lesions can be safely separated from neural structures, complete excision using nerve-sparing techniques may be prioritized. Conversely, when radical excision carries a substantial risk of sensory, motor, sexual, anorectal, or urinary dysfunction, preservation of neural function should be carefully weighed in the surgical decision-making process. Therefore, in patients with suspected direct nerve involvement, management should be individualized according to the extent of neural infiltration, pre-existing neurological deficits, the predominant pain phenotype, and the anticipated functional benefit of surgery [81,82].

The weak correlation between disease extent and pain severity supports the role of sustained neuroinflammatory and neuroplastic changes that maintain pain independently of lesion size or activity [20]. A study by Orr [7] suggests that endometriosis-related pain has a CS component, characterized by sustained hyperexcitability of the central nociceptive system, which can amplify and perpetuate pain perception even after adequate medical or surgical treatment. The authors used the CSI to assess pain perception before and after endometriosis surgery. Higher baseline CSI scores were associated with increased pain severity at postoperative follow-up, despite surgical intervention. Furthermore, patients with multiple chronic overlapping pain conditions, such as fibromyalgia, chronic fatigue syndrome, or migraine, exhibited significantly higher CSI scores after surgery and poorer pain outcomes compared to patients without these comorbidities [7].

Together, these findings underscore the limitations and potential functional consequences of standard surgical approaches in endometriosis-related CPP [1,81]. Extensive laparoscopic pelvic surgery may result in unrecognized nerve injury, contributing to persistent postoperative pain and functional impairment. Consequently, patients are often managed with neuromodulatory or symptomatic treatments without addressing the underlying neural mechanisms of pain. This highlights the importance of a detailed understanding of pelvic nerve anatomy and function, forming the basis of NP, whose objective is to identify, preserve, and, when necessary, appropriately manage pelvic neural structures during surgical interventions [1].

## 6. Discussion

The present review highlights the potential of NP as both a diagnostic framework and a therapeutic strategy for pain management in patients with endometriosis. In this manner, current evidence suggests that NP may serve as a bridge between traditional gynecological assessment, and modern multidisciplinary approaches with a special focus on the understanding of complex pain in endometriosis. The reviewed evidence suggests that a neuropelveological evaluation may facilitate the identification of neuropathic and nociplastic pain mechanisms that frequently remain undetected when using conventional diagnostic approaches. Furthermore, the available evidence supports the concept of endometriosis as a neuroinflammatory condition where neural remodeling and inflammatory mechanisms contribute to persistent pain. The neurobiological perspective reinforces the need to assess patients considering the pain mechanisms surrounding the pathology and integrating them into a routine clinical practice. Additionally, persistent or recurrent pain following conventional treatment when using an excision-based management may lead to repeated interventions as a consequence of residual disease or recurrence of endometriosis. However, the reviewed evidence indicates that in many cases the persistence of pain after medical or surgical management may also reflect peripheral nerve involvement, CS, or nociplastic pain mechanisms [6,71,79,81]. Within this context, NP provides a comprehensive framework that integrates these mechanisms into the assessment and management of endometriosis-associated pain [1,17,83].

A key finding of this review is that the persistence or recurrence of pain following apparently successful surgical treatment reinforces the concept that endometriosis-associated pain cannot be explained solely by the presence or extent of ectopic lesions [5]. This challenges the traditional assumption that endometriosis symptom resolution depends on the complete excision. Consistent with this perspective, accumulating evidence from studies by Song [6], Golińska [57] and Alboni [75] suggest that pain in endometriosis also involves peripheral nerve alterations, neuroimmune interactions, central and peripheral sensitization, all of which may contribute to pain chronification and symptom persistence despite lesion excision.

In this way, the comparison of a series of studies concerning the evaluation of CPP reveals a pattern regarding the presence of persistent pain after medical or surgical management, which should not be interpreted as treatment failure, incomplete resection, or residual disease. Becker [81] noted that the response to medical treatment is heterogeneous and that a significant percentage of patients continue to experience pain despite the use of hormonal therapies. Similarly, various studies have shown that symptoms such as pelvic pain, dysmenorrhea, and dyspareunia may persist or recur even after a technically adequate excision [20,57,74,80,81]. In this context, Orr [7] suggests that a clinical profile consistent with CS, as inferred from CSI scores, may be a relevant factor.

Furthermore, the neuroimaging findings described by Maulitz [59] support the present perspective by identifying structural changes in the brain of patients with endometriosis, suggesting that recurrent visceral pain may be associated with central neuroplasticity processes. The study also emphasizes that the pain experience in endometriosis does not depend exclusively on peripheral pathophysiological mechanisms, but also on central processes related to pain perception, the emotional relationship to pain, and neuroplastic changes associated with chronic pain [59]. Taken together, these findings indicate that persistent pain following conventional treatment may reflect a heterogeneous pain phenotype, in which nociceptive, neuropathic, nociplastic, neuroimmune, and central mechanisms interact [18,25,71]. Consequently, NP recognizes the different pain mechanisms, expanding the traditional paradigm of management by incorporating detailed neuropelvic anatomy, assessment, and imaging into clinical decision making. This approach facilitates the identification of patients who may benefit from surgery or medical treatment alone and those who may require a more comprehensive approach targeting the individual underlying pain mechanisms.

Moreover, the growing recognition of NP is reflected in the recent publication of the first ISON guidelines, which represent the first attempt to standardize the diagnosis and surgical treatment of endometriosis involving sciatic nerve and sacral plexus. These recommendations emphasize the importance of a structured neuropelveological history, neurological examination, functional assessment and a thorough surgical planning. Although these recommendations focus on DIE and remain largely based on expert consensus in specialized centers, they constitute the first international framework to standardize terminology, diagnostic criteria, surgical approaches, and rehabilitation, laying the groundwork for the integration of neuropelveological care into routine clinical practice and for future multicenter studies across diverse healthcare settings [82] (Table 3).

In this regard, pain associated with endometriosis should be understood as a multidimensional experience in which biological, cognitive, and affective components interact. Therefore, procedures aimed exclusively at hormonal suppression, pain management or the resection surgery may not be effective in patients who already exhibit changes related to nociplasticity. Recognizing this distinction is clinically important, as it can help prevent medical overtreatment and surgical reintervention. It can also facilitate earlier referral to multidisciplinary pain management, including pelvic floor rehabilitation, psychological support, and neuropelvic evaluation when neural involvement is suspected [1,2]. Ultimately, integrating NP into routine clinical practice may contribute to a more precise phenotyping of endometriosis-associated pain and improvement of patient care.

## 7. Limitations and Future Perspectives

As previously mentioned, the neuropelveologic approach in endometriosis represents a relatively novel therapeutic strategy and, as such, is associated with several limitations. These include the considerable heterogeneity across studies in study design, surgical techniques, and reported outcomes. Additionally, there remains a paucity of evidence evaluating long-term efficacy and providing a broader perspective on outcomes, including pain, pain recurrence, long-term follow-up, and functional outcomes [2].

The available literature on NP in endometriosis remains insufficient and is largely based on observational studies, retrospective cohorts, and case series, the majority of which are in specialized centers. Allahqoli [2] identified only six studies reporting the efficacy of various neuromodulation techniques in managing endometriosis-related symptoms. Although these findings appear promising, further research is required to more comprehensively establish the benefits and limitations of these approaches. In this way, despite the emergence of novel neuropelveological techniques, the currently available evidence remains limited. This narrative review did not use a predefined systematic search strategy, formal study selection protocol, or risk of bias assessment. This approach may have introduced selection bias and may not have captured all relevant evidence [2]. The findings should therefore be interpreted as a qualitative synthesis rather than an exhaustive assessment of the literature.

Overall, pelvic nerve pathologies, including endometriosis, are frequently misdiagnosed and underestimated due to limited awareness regarding the existence and the underreporting of such conditions. Several factors may contribute to their underrepresentation in clinical practice, including the anatomical complexity of the PNS and the restricted accessibility of pelvic nerves for neurophysiological assessment and neurosurgical interventions. Furthermore, pain is inherently subjective, and despite the availability of validated pain assessment scales, no objective method currently exists to comprehensively characterize or quantify pain in patients with endometriosis. Consequently, discrepancy between symptom perception and disease burden may contribute to a delayed diagnosis, with many patients being identified only after the disease has progressed to more advanced stages [1].

Furthermore, adequate training in surgical techniques is essential to reduce procedural variability and enhance the consistency and reliability of clinical outcomes [75,84]. Additionally, most available data stems from highly specialized centers with extensive experience in retroperitoneal pelvic neuroanatomy and neurofunctional surgery, which limits the reproducibility across centers. This highlights the need for specialized training and adequate infrastructure. Many centers, especially in low and middle-income countries, may lack advanced laparoscopic and neuromodulation equipment required to perform this type of procedure. As a result, NP faces several challenges, including specialized training, surgical complexity, access to surgical technologies, and the economic burden on patients associated with this type of care [82]. To improve clinician awareness, training should include pelvic neuroanatomy, neurological examination, and recognition of symptoms that suggest neural involvement, including cyclical sciatica, dermatomal pain, sensory or motor deficits, and autonomic dysfunction [70,74,82]. Multidisciplinary teams should establish referral pathways for complex cases, while surgeons treating DIE with suspected neural involvement should develop expertise in retroperitoneal pelvic neuroanatomy and nerve sparing surgical principles [63,82].

A 2024 study by Gentles [25] highlighted that, although several tools are currently available to identify CS and nociplastic pain in patients with endometriosis, including semi-objective clinical assessments evaluating pelvic sensitivity, myofascial trigger points, allodynia, hyperalgesia, and pelvic floor pain, these methods have not yet been universally standardized or validated. Nociplastic pain and CS remain particularly difficult to characterize objectively. QST provides a standardized psychophysical approach to assess altered somatosensory processing, but heterogeneous protocols and limited validation currently restrict its clinical use [25]. These limitations complicate pain phenotyping, patient stratification, and comparisons across studies [25,30]. The authors therefore emphasize the need to develop and validate a comprehensive set of diagnostic tools, including neurophysiological assessments, biomarkers, and neuroimaging techniques to enable accurate identification and stratification of pain mechanisms CS in individuals with endometriosis [25]. Standardized and validated assessment methods would not only enhance the reproducibility of research findings but also facilitate their integration into routine clinical practice [7,25].

Despite the clinical potential of NP, several important considerations should be acknowledged. Recently, the ISON guidelines were published; they summarize the current concepts regarding the diagnosis, pathophysiology, and surgical management of endometriosis of the sacral plexus and sciatic nerve. Although the recent publication of said guidelines represents an important step toward the standardization of NP in endometriosis, studies show high heterogeneity in their design, populations, surgical techniques, and outcomes which also restricts direct comparisons between studies [82]. Therefore, the findings presented in this review should be interpreted within the context of these limitations. Studies should focus on validating current diagnostic criteria, surgical indications, long-term outcomes, accessibility and cost-effectiveness in order to be translated across clinical settings. In parallel, advances in artificial intelligence and precision medicine support the idea of multidisciplinary medical management to optimize the stratification of patients, prediction in treatment response and personalized management and rehabilitation. Although these approaches are novel to the field, they represent a promising direction to translate neuropelveological concepts into standardized clinical practice [85]. Future prospective guidelines call for multicenter studies with standardized diagnostic criteria and outcome measures to define the role of NP in the management of endometriosis.

## 8. Conclusions

NP represents a significant conceptual shift in our understanding of pain associated with endometriosis, as it incorporates the role of the peripheral nervous system and subsequent mechanisms of central pain sensitization in the pathophysiology of this disease. The available evidence suggests that pain associated with endometriosis goes beyond the mere presence of ectopic lesions and is due to interactions involving inflammatory, neuroimmunological, and neuroplastic processes that explain the persistence of symptoms even after hormonal or surgical treatment.

Although NP remains an emerging field and current evidence is limited, its integration as a complement to the conventional approach could improve the characterization of different pain phenotypes, optimizing treatment planning by identifying patients who might benefit from specific interventions, such as neuromodulation or neurolysis procedures.

Furthermore, before considering the systematic incorporation of these strategies into clinical practice, well-structured prospective studies are needed to help define therapeutic indications, evaluate long-term functional outcomes, and determine their cost-effectiveness.

In total, NP should not be considered a substitute for current endometriosis treatment, but rather a complementary conceptual and clinical framework that reinforces a multidisciplinary and individualized therapeutic approach based on the possible pain mechanisms involved, with the potential to improve diagnostic accuracy, surgical decision-making, and patients’ quality of life.

## Figures and Tables

**Table 1:** Endometriosis-associated pain phenotypes.

Pain Phenotype	Pathophysiological Mechanism	Associated Clinical Manifestations	Therapeutic Approaches	References
Nociceptive pain.	Activation of peripheral. Nociceptors. Stimulation of nociceptive pathways.	Dysmenorrhoea, dyspareunia, dyschezia, dysuria, and CPP.	Progestogens. Estrogen-progestogen combinations. GnRH agonists. GnRH antagonists.	[18,21,25].
Neuropathic pain.	Lesion or disease affecting the SNS.	Neuropathic symptoms such as radiating pain, paraesthesia, sensory disturbances, and, less frequently, motor deficits.	Neuromodulators: Tricyclic antidepressants. Gabapentin.	[18,21,25].
Nociplastic pain.	Altered nociceptive processing. CS (amplification of pain signaling). Pain hypersensitivity. Enhanced responsiveness of spinal and supraspinal nociceptive pathways.	Pain hypersensitivity, persistent pain, expansion of the painful area, symptoms disproportionate to the visible tissue damage, CPP.	Therapeutic approaches targeting the neurobiological mechanisms involved in pain maintenance. GnRH analogues (hypothetically).	[22,25].

We described the main endometriosis-associated pain phenotypes: nociceptive, neuropathic, and nociplastic pain. Including their underlying pathophysiological mechanism and associated clinical manifestations.

**Table 2:** Pelvic innervation components and their associated clinical manifestations.

Type of Innervation	Involved Structures	Neuroanatomical Territory	Clinical Manifestations	References
Somatic.	Pudendal nerve (ventral rami of S2 to S4). Sacral plexus (lumbosacral trunk and nerve roots S1 to S4).	Perineum. External anal sphincter. External urethral sphincter. Pelvic floor. Portions of the lower extremities.	Entrapment syndromes. Increase the risk of iatrogenic injury during pelvic surgical procedures.	[62,63,64].
Autonomic.	Superior hypogastric plexus. Inferior hypogastric plexus.	Bladder. Rectum. Uterus. Vagina. Pelvic viscera.	Detrusor underactivity. Primary bladder obstruction. Bladder pain syndrome. Irritable bowel syndrome.	[65,66,67,68].
Visceral.	Pelvic visceral afferent and efferent fibers.	Pelvic organs involved in urinary, anorectal, and sexual function.	Neuropathic pain. Incontinence. Visceral dysfunction.	[61,66,69].

Pelvic innervation components, neuroanatomical territories, and associated clinical manifestations.

**Table 3:** Summary of primary clinical evidence on nerve-directed and surgical interventions in endometriosis-associated pain.

Intervention/Topic	Design	n	Outcome	Adverse Events	Evidence Level/Risk of Bias	Study
Standard excisional surgery + CSI assessment	Prospective cohort	NS	Higher preoperative CSI associated with worse postoperative pain; 23% underwent second surgery at 7-yr follow-up	NR	Observational cohort, moderate risk of bias (no comparator, CSI not a direct CS measure)	[7]
Standard surgical management	Retrospective cohort	NS	44.6% underwent second surgery	NR	Observational cohort, moderate–high risk of bias.	[80]
Standard excisional surgery	Retrospective cohort	345	28.1% (97/345) persistent postoperative pain despite no detectable lesions	NR	Observational cohort, moderate risk of bias.	[75]
Surgery + medical therapy	Systematic review	-	Median 31% persistent pain at the end of medical treatment.	NR	Systematic review of heterogeneous observational studies, moderate risk of bias.	[81]
Neuromodulation techniques.	Narrative synthesis of 6 studies.	NS	Described as “promising”.	NR	Small evidence base, individual study quality not detailed, low–moderate confidence.	
Diagnosis and surgical management of sciatic/sacral plexus DIE. (includes neurolysis)	Expert consensus guideline.	N/A	Standardizes terminology, indications, technique.	N/A	Expert consensus, not primary clinical data.	

NS: Not specified, NR: Not Reported, N/A: Not applicable.

## Data Availability

No new data were created or analyzed in this study. Data sharing is not applicable to this article.

## Abbreviations

The following abbreviations are used in this manuscript:

NP	Neuropelveology
PNS	Pelvic nervous system
CPP	Chronic pelvic pain
CSI	Central Sensitization Inventory
DIE	Deep infiltrating endometriosis
CNS	Central nervous system
CPPS	Chronic pelvic pain syndrome
CS	Central sensitization
TVUS	Transvaginal ultrasound
MRI	Magnetic resonance imaging
VEGF	Vascular Endothelial Growth Factor
NGF	Nerve Growth Factor
BDNF	Brain-Derived Neurotrophic Factor
QST	Quantitative sensory testing
